# Hypoxia inducible factor-1 mediates the expression of the immune checkpoint HLA-G in glioma cells through hypoxia response element located in exon 2

**DOI:** 10.18632/oncotarget.11628

**Published:** 2016-08-26

**Authors:** Layale Yaghi, Isabelle Poras, Renata T. Simoes, Eduardo A. Donadi, Jörg Tost, Antoine Daunay, Bibiana Sgorla de Almeida, Edgardo D. Carosella, Philippe Moreau

**Affiliations:** ^1^ Commissariat à l'Energie Atomique et aux Energies Alternatives, Institut des Maladies Emergentes et des Thérapies Innovantes, Service de Recherches en Hémato-Immunologie, Hôpital Saint-Louis, Paris, France; ^2^ Université Paris-Diderot, Sorbonne Paris-Cité, UMR E5, Institut Universitaire d'Hématologie, Hôpital Saint-Louis, Paris, France; ^3^ Lebanese University, School of Medicine, Hadath, Lebanon; ^4^ Instituto de Ensino e Pesquisa da Santa Casa de Belo Horizonte, IEP/SCBH, Belo Horizonte, Minas Gerais, Brasil; ^5^ Divisão de Imunologia Clínica, Departamento de Clínica Médica, Faculdade de Medicina de Ribeirão Preto, Universidade de São Paulo, Ribeirão Preto, São Paulo, Brasil; ^6^ Centre d'Etude du Polymorphisme Humain, Fondation Jean-Dausset, Laboratory for Functional Genomics, Paris, France; ^7^ Commissariat à l'Energie Atomique et aux Energies Alternatives, Centre National de Genotypage, Laboratory for Epigenetics and Environment, Evry, France

**Keywords:** HLA-G, HIF-1, exon 2 HRE, glioma

## Abstract

HLA-G is an immune checkpoint molecule with specific relevance in cancer immunotherapy. It was first identified in cytotrophoblasts, protecting the fetus from maternal rejection. HLA-G tissue expression is very restricted but induced in numerous malignant tumors such as glioblastoma, contributing to their immune escape. Hypoxia occurs during placenta and tumor development and was shown to activate *HLA-G*. We aimed to elucidate the mechanisms of HLA-G activation under conditions combining hypoxia-mimicking treatment and 5-aza-2′deoxycytidine, a DNA demethylating agent used in anti-cancer therapy which also induces *HLA-G*. Both treatments enhanced the amount of *HLA-G* mRNA and protein in HLA-G negative U251MG glioma cells. Electrophoretic Mobility Shift Assays and luciferase reporter gene assays revealed that *HLA-G* upregulation depends on Hypoxia Inducible Factor-1 (HIF-1) and a hypoxia responsive element (HRE) located in exon 2. A polymorphic HRE at −966 bp in the 5′UT region may modulate the magnitude of the response mediated by the exon 2 HRE. We suggest that therapeutic strategies should take into account that HLA-G expression in response to hypoxic tumor environment is dependent on *HLA-G* gene polymorphism and DNA methylation state at the *HLA-G* locus.

## INTRODUCTION

Hypoxia is a key micro-environment factor in aggressive tumors that induces angiogenesis to supply cells with oxygen and nutrients. Therapies using antiangiogenic drugs have thus been developed to stop tumors from growing their own blood vessels. Nonetheless, it is reported that antiangiogenic therapy induces acute hypoxic stress, leading to tumor invasion and metastasis because of the acquisition of compensatory mechanisms, which could select specific tumor cell populations able to grow and proliferate in low oxygen environment [1]. In tumor cells, hypoxia was previously shown to induce expression of *HLA-G* gene [2, 3], an immune checkpoint gene whose function is involved in immune evasion [4]. Otherwise, the use of DNA methylation inhibitors such as 5-aza-2′deoxycytidine (5-aza-dC; decitabine) has been proposed in cancer therapy [5–9] and was demonstrated to induce HLA-G expression *in vitro* [10, 11]. Therefore, hypoxia environment combined with anticancer therapy might favor HLA-G expression and consequently tumor progression. The identification of molecular mechanisms implicated in the *HLA-G* induction in these conditions is of crucial importance in the scope of developing future anti-cancer therapies and more particularly immunotherapies.

Human leukocyte antigen G (HLA-G) is a non-classical HLA class I molecule of the major histocompatibility complex [12]. It exhibits low polymorphism [13], and restricted tissue distribution in placenta [14] and immuno-privileged sites such as thymus and cornea [15, 16]. Alternative splicing of the primary transcript leads to 7 mRNAs that can be translated into 7 protein isoforms, membrane bound (G1 to G4) and soluble (G5 to G7) forms [17–19]. HLA-G isoforms regulate the immune responses by modulating the function of natural killer cells, cytotoxic T lymphocytes and antigen presenting cells, through at least two inhibitory receptors IL-T2 [20, 21] and IL-T4 [22]. More particularly, HLA-G induces T regulatory cells (Tregs) and regulatory antigen presenting cells (APCs) that modulate immune response and promote tolerance [23]. Recently, evidence was given that the binding of HLA-G to IL-T2 receptor suppresses B cell responses [24]. Physiologically, HLA-G plays a key role in maintaining immune tolerance at the fetal-maternal interface and has been associated with a lower risk of development of acute and chronic rejection in allograft transplantation [25–27]. This immune checkpoint molecule is also expressed in numerous tumor types, contributing to their immune evasion [28], and its expression has been correlated with poor clinical outcome of patients [11, 28–32]. In agreement, using a xenotumor model or syngeneic tumor cells in mice, it has been reported that HLA-G is involved in tumor immune surveillance *in vivo* [33, 34]. Therefore, regarding the growing and successful use of antibodies against checkpoints such as anti-CTLA-4 and anti- PD-L1/PD-1 to restore antitumor immunity [35], HLA-G appears as a wider and promising relevant target for cancer immunotherapy [12].

Nonetheless, despite the accumulation of data on the identification of elements controlling HLA-G expression most of them are still to be described. They differ from those of classical HLA class I gene since almost all known regulatory sequences for these genes are disrupted in the *HLA-G* promoter region [36]. In fact, it is demonstrated that HLA-G gene can be regulated by specific regulatory sequences such as Enhancer L at −12 kb from ATG [37], LINE-1 at −4 kb [38] and LCR at −1.2 kb [39], and specific transcription factors such as CREB1/ATF [40], RREB1[40–42], HSF1[42], IRF1[43] and PR [44]. In addition, we and others have demonstrated the modulation of *HLA-G* expression by epigenetic mechanisms (DNA methylation and histone modifications) [10, 45], and micro-environmental factors [46–50]. Particularly, *HLA-G* can be induced by hypoxia [51, 52] or hypoxia-mimicking conditions as shown previously with melanoma cell lines cultured in the presence of desferrioxamine (DFX) [2, 53].

Hypoxia is a physiologically relevant tumor-related stress defined by a decrease of cellular oxygen concentrations which in turn leads to an adaptive response modulating the expression of genes involved in angiogenesis, erythropoiesis and glycolysis [54]. The signal transduction in hypoxic cells is mainly conducted by the basic helix-loop-helix bHLH/PAS hypoxia inducible factor (HIF) [55, 56] comprising a labile HIF-α subunit (HIF-1α and HIF-2α isoforms) whose stability is regulated by an oxygen-dependent hydroxylation, and a stable HIF-β subunit. Under hypoxia or hypoxia-mimicking (DFX) conditions, stabilized HIF-1α translocates into the nucleus where it binds HIF-1β. The α/β heterodimeric transcription factor then recognizes hypoxia responsive elements (HREs) present on target genes causing activation of transcription [57]. Regarding *HLA-G*, a consensus HRE has been identified by *in silico* analysis at −242 bp upstream of the ATG target site [53]. Nonetheless, whether or not this site is involved in the DFX-induced HLA-G transcription in melanoma cells [2, 53], is still unsolved.

Moreover, we recently demonstrated that HLA-G expression may be upregulated in grade IV glioblastoma [11], a very aggressive tumor in which hypoxic microenvironment plays a key role in the disease progression [58]. We also reported that 5-aza- dC demethylating treatment induced HLA-G expression in a cellular model of human glioblastoma, the HLA-G negative U251MG glioma cell line [11]. Since melanocytes and glial cells are both derived from similar embryological origin, and their malignant transformed counterparts share many biological properties and express common tumor markers, we thus investigated U251MG cell line for the induction of HLA-G expression and the associated regulatory mechanisms driven by hypoxia-mimicking conditions alone or combined with 5-aza-dC.

## RESULTS

### *HLA-G* expression in U251MG glioma cells is induced by hypoxia-mimicking DFX and is upregulated by DFX combined with DNA demethylating treatment

**Figure 1:**
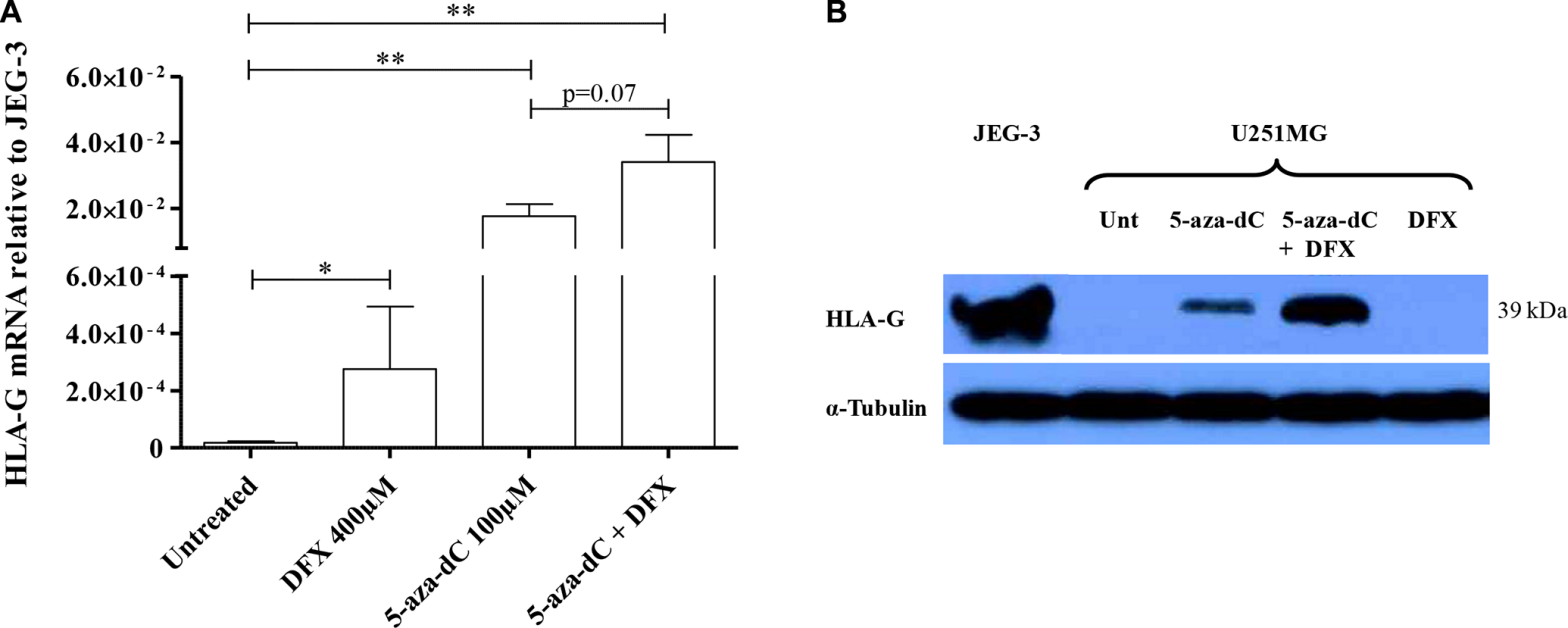
Hypoxia-mimicking conditions induce HLA-G expression in HLA-G negative glioblastoma cell line U251MG (**A**) Real-time RT-PCR analysis, targeting all *HLA-G* mRNA forms, carried out on cells cultured with normal growth conditions or treated with DFX at 400 μM for 24 h, 5-aza-2′-deoxycytidine (5-aza-dC) at 100 μM for 72 h, or 5-aza-dC combined to DFX (3 independent experiments in duplicates). The cells exposed to 5-aza-dC exhibit a ~103 mean-fold increase in *HLA-G* transcript level, while combined treatments give an additional ~2 mean-fold increase in *HLA-G* transcript level (3 independent experiments in duplicates). Data is presented as mean ± SEM and statistical analysis was performed using Mann-Whitney *U* tests (* indicates a *p* value < 0.05 and ** indicate a *p* value < 0.01). (**B**) Representative western blot analysis of HLA-G activation revealed with 4H84 mAb (2 independent experiments). U251MG cells were either treated with conditions described above or not (Unt: untreated). α-Tubulin was used as an internal control. JEG-3: choriocarcinoma cell line expressing HLA-G.

We first investigated the effect of hypoxia-mimicking agent desferrioxamine (DFX) treatment on *HLA-G* expression in U251MG glioma cells (Figure 1A and Supplementary Figure S1). We observed a significant (*P* < 0.05) upregulation of *HLA-G* gene transcription suggesting that HIF could be involved in the activation of *HLA-G* expression. Giving that demethylating agent 5-aza-dC has been proposed for anti-tumoral therapy, and having previously demonstrated that DNA demethylation is crucial for HLA-G expression, we hypothesized that CpG methylation in HIF target sites (5′-RCGTG-3′) could moderate the observed *HLA-G* mRNA induction. We thus exposed cells to the demethylating agent 5-aza-dC, using standard conditions, at 100 μM for 72 hours, and then to DFX at 400 μM for an additional 24 hours. Real-time RT-PCR analysis revealed a strong upregulation of *HLA-G* transcripts following 5-aza-dC treatment alone, and although not significant (*P* = 0.07) an additional 2× mean-fold enhancement of *HLA-G* transcript levels was observed following both 5-aza-dC and DFX treatments (Figure 1A). In the latter condition, we also observed by Western blot analysis the upregulation of HLA-G protein expression (Figure 1B). Therefore, hypoxia-mimicking microenvironment may be a pertinent parameter participating in the *HLA-G* gene activation and in the enhancement of *HLA-G* gene expression when DNA demethylation is occurring.

### HIF-1 is involved in *HLA-G* gene expression under hypoxia-mimicking conditions

**Figure 2:**
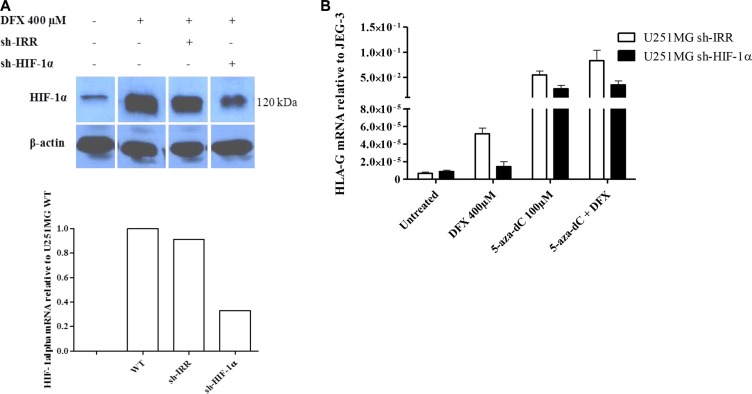
HIF-1 is implicated in the activation of HLA-G expression in hypoxia-mimicking conditions (**A**) Representative western blot and real-time RT-PCR analysis of HIF-1α expression in U251MG wild type cells (WT), or transfected with an irrelevant shRNA (sh-IRR) or HIF-1α specific shRNA (sh-HIF-1α). Cells were treated with DFX (400 μM) for 3 h. Western blot was performed with cytoplasmic extracts and β-actin as an internal control. HIF-1α and β-actin bands were collected from the same gel to assemble the picture. qRT-PCR results were compared to those of U251MG WT (assigned a value of 1). (**B**) Real-time RT-PCR analysis of *HLA-G* expression in transfected U251MG cells (U251MG sh-IRR and U251MG sh-HIF-1α; 2 independent experiments in duplicates for each transfectant), either treated with 5-aza-dC (100 μM) for 72 h and DFX (400 μM) for 24 h, or not. Results were compared to those of HLA-G positive cell line JEG-3 (assigned a value of 1).

In order to investigate the role of HIF in the upregulation of *HLA-G* gene expression under hypoxia-mimicking microenvironment we transfected U251MG cells either with a control shRNA (sh-IRR) or with a shRNA directed against the α subunit of the transcription factor (sh-HIF-1α). Western blot analysis performed with the wild type cells or the control shRNA transfectant showed HIF-1α stabilization at 3 h following DFX treatment. On the contrary, HIF-1α was reduced both at the transcriptional and the protein expression levels following DFX treatment in cells transfected with sh- HIF-1α (Figure 2A). Then, to test the effect of HIF-1 on *HLA-G* gene transcriptional activity, we performed real time RT-PCR analysis on the control shRNA transfectant and the HIF-1α shRNA transfectant, both exposed to DFX and/or the demethylating agent 5-aza-dC using the same conditions as above. Under hypoxia-mimicking conditions, amounts of *HLA-G* gene transcripts produced by the sh-IRR transfectant were not achieved with the sh-HIF-1α transfectant (Figure 2B), either in the absence or the presence of 5-aza-dC in the culture medium (Figure 2B). These results support that the upregulation of *HLA-G* transcription in cells cultured in the presence of DFX involves HIF-1.

### The 1.4 kb-5′ untranslated regulatory region (5′UTR) of *HLA-G* gene contains ineffective HIF-1 target sites

**Figure 3:**
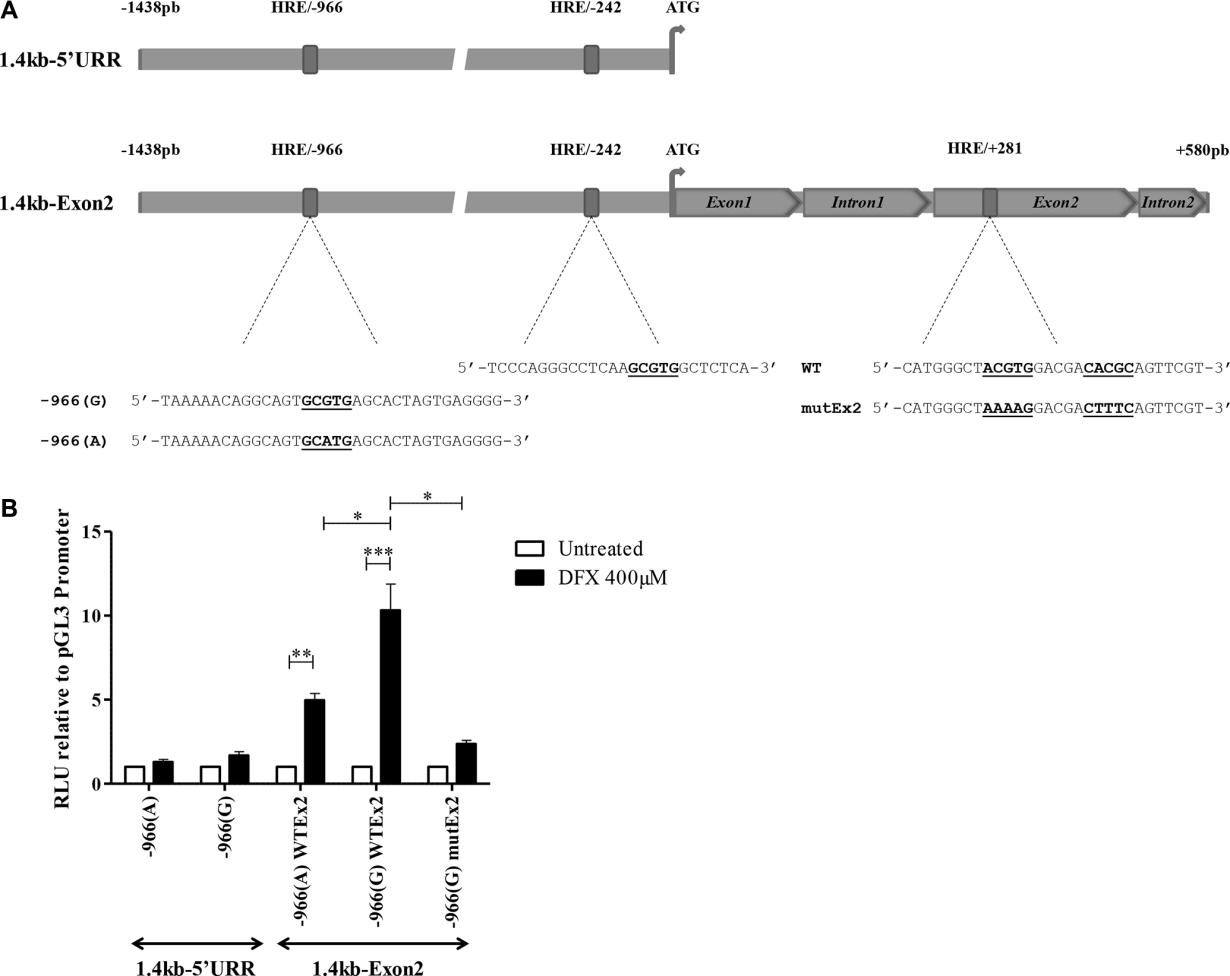
HIF-1α activates HLA-G expression through HREs located on the *HLA-G* locus (**A**) Schematic representation of the location of HREs in the 1.4 kb-5′URR and 1.4 kb-Exon2 fragments of *HLA-G* cloned in pGL3 basic vector upstream of the firefly luciferase gene. The sequence of each HRE is described. −966 HBS contains a natural G/A polymorphism indicated as −966(G) and −966(A). +281 HRE contains a double HBS that was cloned either in its wild type (WT) or double mutated (mutEx2) forms. (**B**) Luciferase activity assayed with U251MG transfected with either constructs, treated or not with DFX (400 μM) for 24 h. Results were normalized to luciferase activity in U251MG cells transfected with pGL3 Promoter vector (Promega), and compared to luciferase activity in untreated cells (assigned a value of 1). A co-transfected pRL-TK Renilla Luciferase reporter vector served as internal control for transfection efficiency and extract preparation. Data are presented as mean ± SEM of 3 independent experiments in duplicates, and statistical analysis was performed using Wilcoxon matched-pairs signed rank test (* indicates a *p* value < 0.05, ** indicate a *p* value < 0.01 and *** indicate a *p* value < 0.001).

*In silico* analysis of the 1.4 kb *HLA-G* region upstream to the ATG translation initiation site revealed two potential HREs containing the hypoxia binding site (HBS) 5′-RCGTG- 3′ at positions −242 bp (5′-TCCCA GGGCCTCAAGCGTGGCTCTCA-3′) and −966 bp (5′-TA AAAACAGGCAGTGCGTGAGCACTAGTGAGGGG-3′) (Figure 3A). Interestingly, the −966 HBS contains a natural A/G polymorphism (−966(A)/−966(G)) located at the 5′end of the site [59].

To assess the functionality of these putative HIF target sites, we first analyzed the activity of the 1.4kb-5′UTR variant having an intact −966 HBS (G form) in U251MG cells. The fragment was subcloned upstream of the firefly luciferase reporter gene into the promoterless pGL3 basic vector (Figure 3A). Extracts from transfected cells, subjected or not to DFX treatment during 24 h, were analyzed for luciferase activity. We observed that the activity of the 1.4 kb-5′UTR was slightly affected by DFX treatment and was not statistically significant, suggesting that HREs in the 1.4 kb *HLA-G* promoter region are ineffective (Figure 3B).

**Figure 4:**
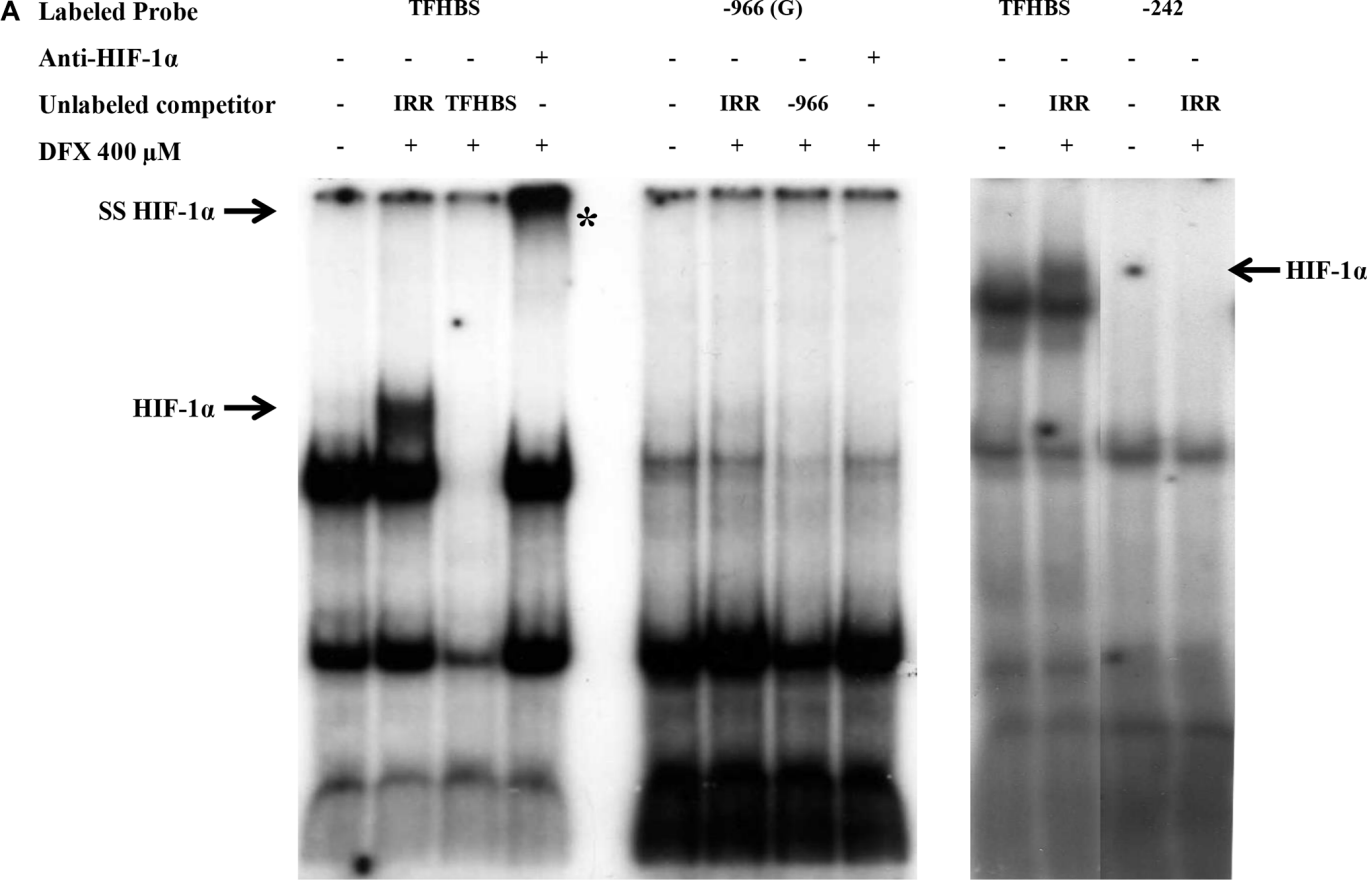

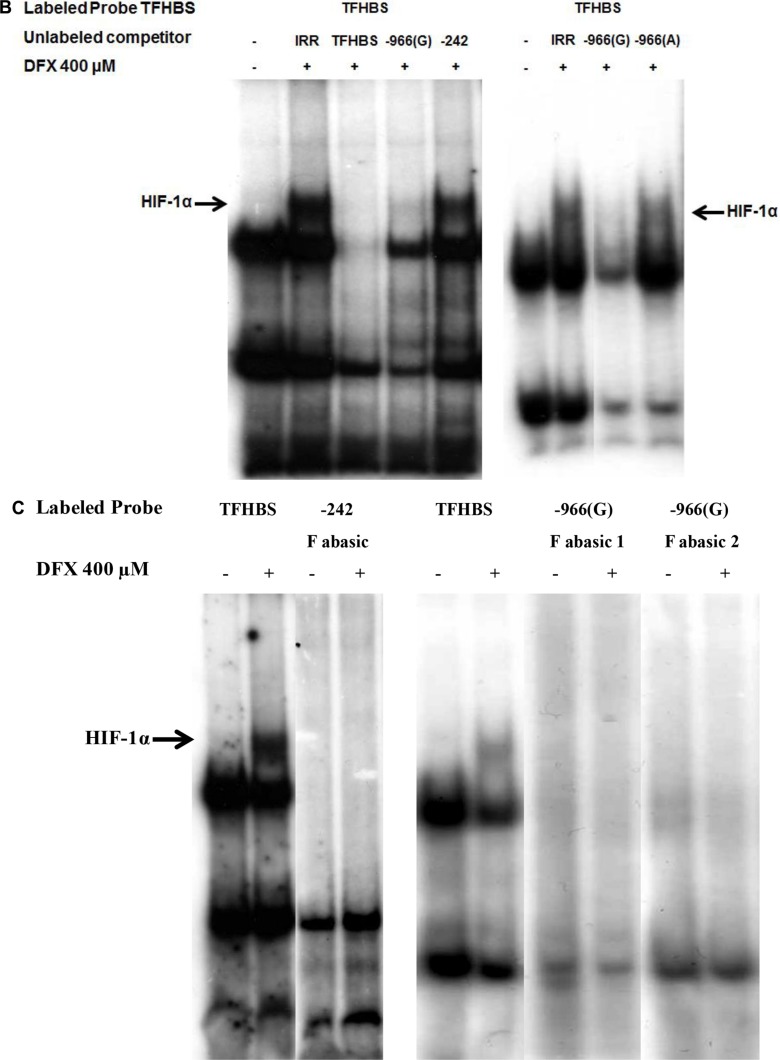
Absence of HIF-1α binding on −966 and −242 HREs of *HLA-G* promoter in EMSA performed with nuclear extracts of U251MG cells treated (+) or not (−) with DFX (400 μM) for 3 h Radiolabeled probe TFHBS (*transferrin* gene HBS) was used as a positive control for HIF-1α binding. (**A**) EMSA performed with radiolabeled probes −966(G) and −242. Competition experiments were carried out by adding a 100-fold molar excess of unlabeled double stranded irrelevant (IRR) or cold specific probe. HIF-1α is demonstrated by supershift analysis using nuclear extracts incubated overnight with an anti-HIF-1α antibody prior to binding experiments. The supershifted complex is indicated by «SS HIF-1α» and «*». (**B**) EMSA performed with radiolabeled probe TFHBS and 100-fold molar excess of unlabeled competitors. −966(A) is the mutated form of −966(G) HBS. (**C**) EMSA performed with radiolabeled probes containing abasic sites: −242 F abasic; −966 (G) F abasic 1; −966 (G) F abasic 2 (see Material and methods). The HIF-1α/Probe complex is indicated by an arrow «HIF-1α» on the gel.

We also performed EMSA using double stranded ^32^P labeled oligonucleotides, containing putative *HLA-G* −242 HBS or −966(G) HBS (Figure 4A). The HIF- 1 stabilization and binding was controlled using a ^32^P labeled oligonucleotide containing HRE sequence from the *transferrin* gene promoter (TFHBS), and nuclear protein extracts pre-incubated with an anti-HIF-1α antibody for supershift experiments. In agreement with luciferase experiments, we noted that these two *HLA-G* HREs were unable to bind HIF-1 while a HIF-1/TFHBS complex was observed (Figure 4A). We also performed competition experiments with a ^32^P labeled TFHBS oligonucleotide in the presence of an excess of cold *HLA-G* −242 or −966(G) oligonucleotides. The −242 oligonucleotide did not alter the complex, thus confirming that this *HLA-G* HRE is nonfunctional. However, we observed that an excess of −966(G) oligonucleotide altered the HIF-1/TFHBS complex, suggesting a slight affinity to HIF- 1 in comparison to −242 HBS, but not strong enough to transactivate *HLA-G* promoter in luciferase assays (Figure 4B).

Finally, it is known that hypoxia responsive elements are prone to oxidative damage to DNA, leading to the formation of an abasic site in one strand, with especially high sensitivity to oxidative damage of the terminal guanines in the HIF-1 DNA recognition sequence [60, 61]. Since a greater binding affinity of HIF-1 to these abasic sites was previously observed *in vitro,* we generated abasic sites for −242 or −966(G) oligonucleotides. EMSA, performed with these oligonucleotides incubated with nuclear extracts of U251MG cells treated or not with DFX during 3 h, did not reveal any HIF-1 binding (Figure 4C). This strongly suggests that efficient HIF target sites at the *HLA-G* locus, if any, are located outside the 1.4kb promoter sequence.

### HIF-1 transactivates *HLA-G* gene through a HRE located in exon 2

In an attempt to identify functional HIF target sites along the *HLA-G* locus, we focused on a putative HRE we localized by *in silico* analysis in the exon 2 (Figure 3A). The sequence contains two HBSs at positions +281 bp (sens) and +291 pb (antisens) from ATG. To investigate this putative HRE, we performed luciferase assays with U251MG cells transfected with the promoterless pGL3 basic vector, in which we inserted a DNA fragment covering both the 1.4kb-5′UTR and a sequence spanning ATG to position +580 pb at the beginning of intron 2 of *HLA-G* gene (1.4 kb-Exon2 construction) (Figure 3A). The level of luciferase activity observed with extracts from U251MG cells transfected with 1.4 kb-Exon2 and treated with DFX was significantly enhanced in comparison to the level of luciferase activity of untreated transfected cells (Figure 3B). These results strongly argue for a major role of this region in the DFX response. Interestingly, a 5 mean-fold enhancement was observed in the presence of −966(A) SNP and a 10 mean-fold enhancement in the presence of −966(G) SNP. Next, using a mutated 1.4 kb-Exon2 fragment in which the two HBSs at +281 bp and +291 bp have been scrambled, we showed that mutations impaired the upregulation of luciferase activity, thus demonstrating that exon 2 HRE is a major target for factors induced by hypoxia mimicking DFX (Figure 3B).

**Figure 5:**
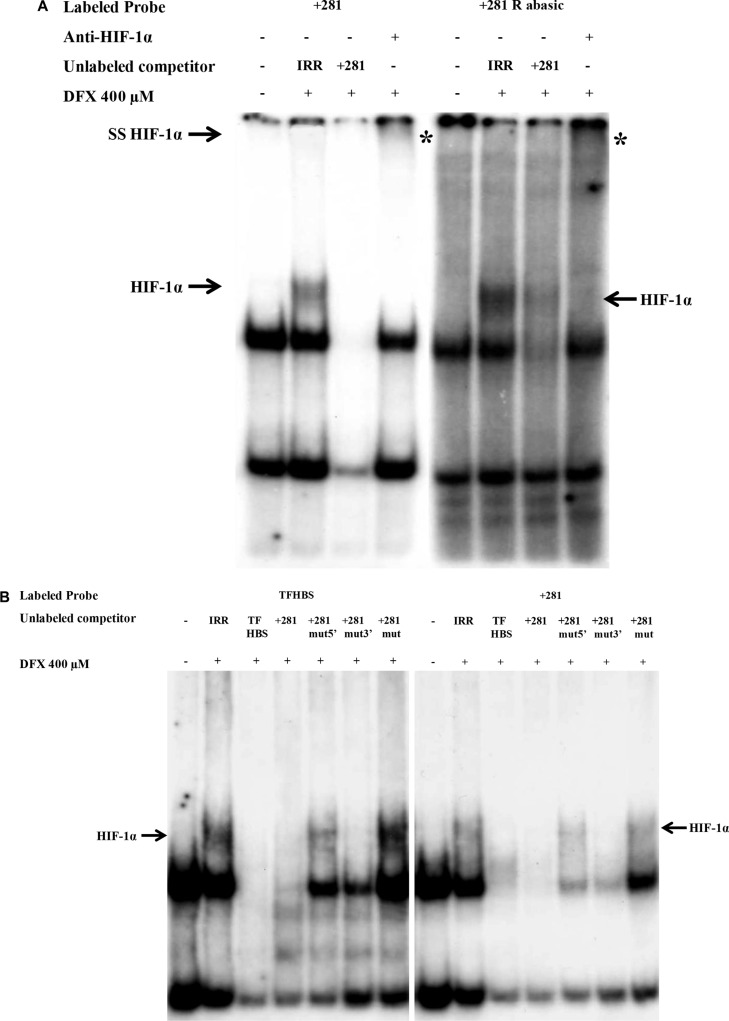

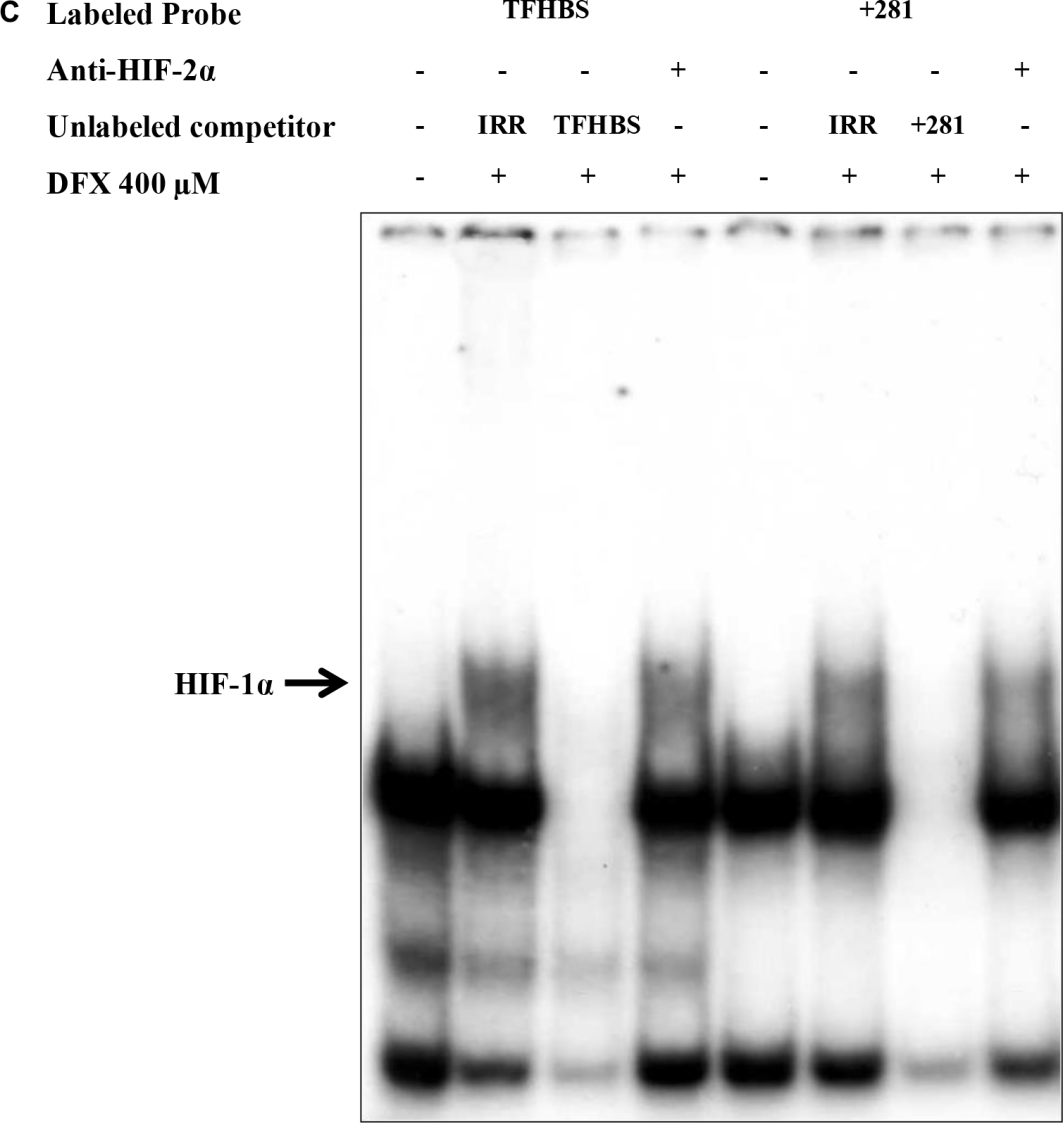
EMSA validation of HIF-1α binding to the HRE located in exon 2 of *HLA-G* gene (+281) with nuclear extracts of U251MG cells treated (+) or not (−) with DFX (400 μM) for 3 h **A**) EMSA performed with radiolabeled *HLA-G* probes containing +281 HRE with 2 HBS (5′ and 3′), either wild type (+281) or abasic (+281 R abasic). Competition experiments were realized by adding a 100- fold molar excess of unlabeled double stranded irrelevant or specific cold probe. The HIF-1α/Probe complex is indicated by an arrow «HIF-1α». Nuclear extracts were also incubated overnight with an anti-HIF-1α antibody prior to binding experiments. The supershifted complex is indicated by an arrow «SS HIF-1α» and «*». (**B**) EMSA performed with radiolabeled control probe TFHBS (*Transferrin* gene HBS) and +281 HRE in the presence of a 100-fold molar excess of unlabeled competitors: IRR (irrelevant), TFHBS, +281, +281 mut5′ (5′ HBS mutated 5′-CATGGGCTAAAAGGACGACACGCAGTTCGT-3′), 281 mut3′ (3′ HBS mutated 5′-CATGGGCTACGTGGACGACTTTCAGTTCGT-3′) and +281 mut (3′ and 5′ HBS mutated 5′-CATGGGCTAAAAGGACGACTTTCAGTTCGT-3′). (**C**) EMSA performed with TFHBS and +281 probe with nuclear extracts incubated overnight with anti-HIF-2α antibody prior to binding experiments.

To further investigate that *HLA-G* HRE in exon 2 was a direct target for HIF, we first carried out EMSAs with nuclear extracts of U251MG cells exposed or not to DFX during 3 h and ^32^P-labeled double stranded oligonucleotide containing +281 HBS and +291 HBS (+281 HRE). Incubation of nuclear extracts of cells exposed to DFX with the labeled +281 HRE oligonucleotide revealed a specific complex which was absent when nuclear extracts of untreated cells were used (Figure 5A). This complex disappeared after adding an excess of cold wild type +281 HRE oligonucleotide, and was shifted with an anti-HIF-1α antibody. We also performed experiments with +281 HRE oligonucleotide containing abasic HBSs that generated a DFX-induced specific complex, exhibiting a greater intensity than the wild type sites (Figure 5A). Competition experiments conducted with mutated +281 HRE in which the two HBS have been scrambled did not alter the pattern. In contrast, the competition was partial in the presence of an excess of cold oligonucleotides in which only one of the HBS was mutated (Figure 5B). Finally, supershift experiments performed with nuclear extracts of DFX-treated cells, incubated with ^32^P-labeled +281 HRE in the presence of anti-HIF-1α and anti-HIF-2α antibodies, identified only the presence of HIF-1α in the complex (Figure 5C).

**Figure 6:**
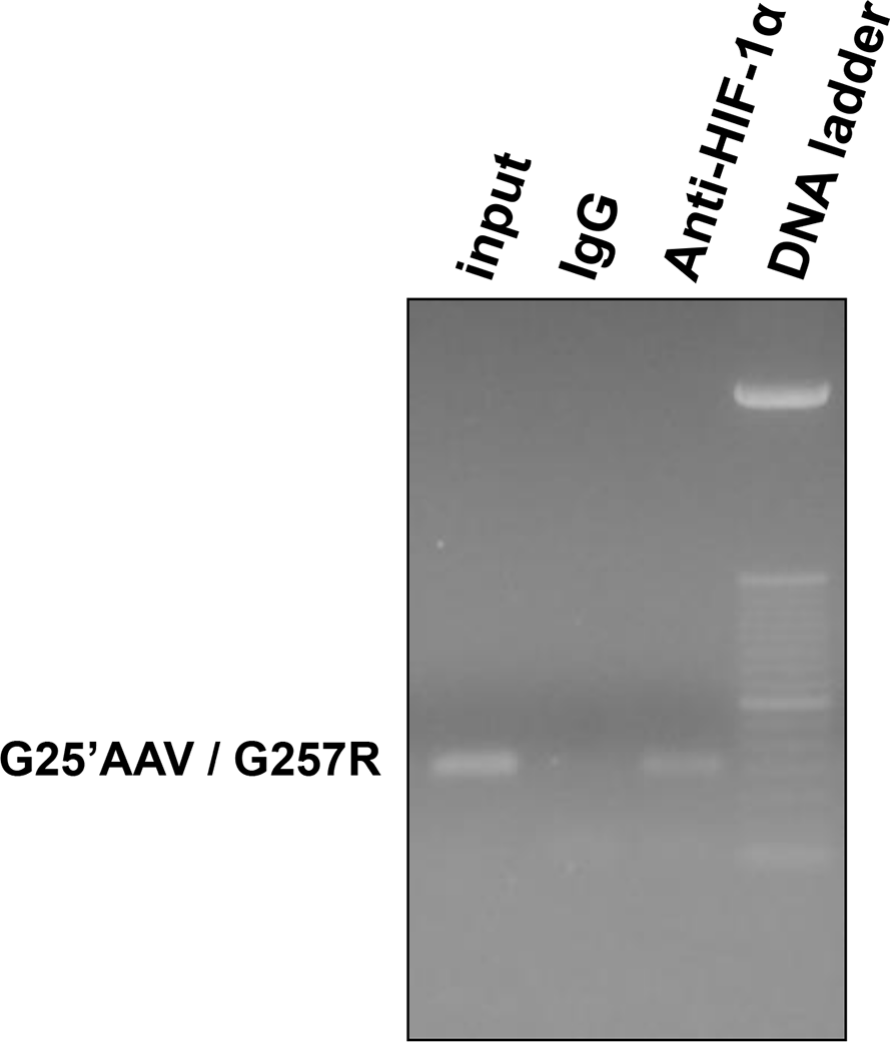
HIF-1 binding to the *HLA-G* gene in U251MG cells treated with 100 μM 5-aza-dC (72 hours) and 400 μM DFX (additional 3 hours) ChIP experiment performed with anti-HIF-1α antibody or control Rabbit IgG. G25′AAV / G257R indicates primer set used for PCR targeting exon 2 of *HLA-G* gene; Input indicates input chromatin used as PCR control.

Additionally, we analyzed the HIF-1 binding to the *HLA-G* locus by ChIP experiments on U251MG cells. PCR targeting *HLA-G* exon 2 revealed that stabilized HIF-1 was positioned at this chromatin region of the *HLA-G* locus when cells were cultured with conditions allowing both HIF-1α stabilization (DFX during 3 h) and high level of *HLA-G* gene induction (5-aza-dC treatment) (Figure 6).

Altogether, these results demonstrate that HRE in exon 2 of *HLA-G* gene is a functional target site for HIF-1 in response to hypoxia-mimicking conditions, and strongly suggest that −966(G) SNP may influence the level of the response.

### *HLA-G* HBS in U251MG cells are differentially methylated

Having demonstrated the impact of demethylating agent in the enhancement of *HLA-G* transcriptional activity in DFX-treated U251MG cells, we finally analyzed the methylation status of *HLA-G* HREs using primer sets AMP1 and AMP2 targeting 5′ UTR, and primer set AMP3 targeting exon 2. Sodium bisulfite conversion and pyrosequencing of DNA from U251MG cells (2 independent experiments) revealed 100 % conversion of cytosines located outside CpG dinucleotide context (data not shown). Notably we observed that the percentage of CpG methylation was 91% at +281 HBS, 66.5% at +291 HBS (+281 HRE) and 91.4% at −966 HRE (Table 1). Lower CpG methylation at + 291 HBS is thus in agreement with HIF-1-mediated gene activation in cells treated with DFX alone. In addition these results support that demethylation treatment is likely to favor HIF-1 binding following DFX treatment.

**Table 1:** *HLA-G* HBS methylation in U251MG cells analyzed by pyrosequencing of bisulfite treated DNA (mean of 2 independent experiments)

HLA-G_AMP1 ( −966 HBS) (Nb) CpG1 (%) 91.4																
**HLA-G_AMP2 ( −242 HBS)**																
**(Nb)**	CpG1	CpG2	CpG3	CpG4	CpG5	CpG6	CpG7									
(%)	96	89	81.5	**86.5**	91	52	84.5									
**HLA-G_AMP3 ( +280 HBS and +290 HBS)**																
**(Nb)**	CpG1	CpG2	CpG3	CpG4	CpG5	CpG6	CpG7	CpG8	CpG9	CpG10	CpG11	CpG12	CpG13	CpG14	CpG15	CpG16
(%)	92	97	95.5	99	95	90.5	80	100	96.5	**91**	100	**66.5**	87	81.5	97	61.5
CpG17	CpG18	CpG19	CpG20	CpG21	CpG22	CpG23	CpG24									
96.5	82.5	34.5	61	53	82.5	84.5	87									

Percentage of methylation (%) and number of CpG (Nb) analyzed for each amplicon from 5′ to 3′. Bold and underlined values correspond to CpG located within the *HLA-G* HBS.

## DISCUSSION

HLA-G immune checkpoint plays a role in maintaining immune tolerance at the fetal-maternal interface, and was shown to participate in tumor immune escape [4, 33]. Hypoxia is a common micro-environmental factor to these major contexts of HLA-G expression [62, 63]. We here demonstrate the induction of HLA-G in glioma cells under hypoxia-mimicking conditions and, for the first time, a functional HIF-1 target site. It is noteworthy that binding of HIF-1 to a transcriptionally active HRE was previously demonstrated for the gene encoding another checkpoint molecule, PD-L1 [64], suggesting that a close interaction between hypoxia and immune regulation exits. Although the amount of HLA-G transcription and protein expression was predominantly controlled by DNA demethylation processes, we also observed that the magnitude of HLA-G expression was still boosted by superimposing hypoxia-mimicking conditions. Therefore, the present work and previous ones, demonstrating that *HLA-G* gene expression in melanoma cells is induced by hypoxic conditions [2, 53], clearly support the contribution of this micro-environment factor in the expression and the level of HLA-G expression. Hypoxia-mimicking conditions were also shown to activate factors associated with extracellular matrix degradation and invasion of glioma cells *in vitro* [65, 66]. Moreover, hypoxia was recently demonstrated to induce *in vivo* macrophage polarization and re-education toward an M2 phenotype in U251MG glioblastoma model [67]. Therefore hypoxia appears as a key factor in the processes that could contribute to glioma evasion.

Unexpectedly, luciferase assays and EMSA experiments revealed that despite the identification of 2 putative HREs in the 5′ UTR, *HLA-G* gene induction occurred more particularly through the HRE located in exon 2. It is a fact that regulatory elements located within the transcribed portions of genes are common, but such elements in coding exons are much less frequent. Some of the documented examples include c-myc (exon 1) [68], rabbit α-globin (fragment extending from exon 1 to intron 2) [69] and keratin-18 (exon 6) [70], and concern both positive and negative regulatory elements. Regarding classical *HLA* class I genes, a negative regulatory element has been described in exon 1 [71]. Moreover, a large number of potential high affinity HIF-binding sites have been identified across the genome in promoter regions, exons and introns, and many remote from known promoters [72–74]. Demonstration has also been made that HIF DNA binding can have an effect on target gene regulation over great distances, and that the hypoxia-inducible gene expression was qualitatively and quantitatively similar, independently of the position of HIF binding on the gene locus [72].

Many studies have characterized the robustness of HIF binding sites. In the binding regions, the A predominates over G at the R position of the core 5′-RCGTG-3′ motif, and there are HIF sequence preferences beyond the HBS. Indeed, HIF binding regions are GC rich, and there is a HIF preference for T at −1 bp of the R position and for a C or G at −2 bp of the R position [72, 75]. It is remarkable that none of these preferences are observed for −242 putative HBS, which could explain the lack of HIF binding to this region in EMSA. On the other hand, all the requirements are met in the HRE we identified in the exon 2 and also at −966 bp. In addition, unlike the −966(G), the +281 HRE region contains a double HBS in close vicinity to each other, which may explain the great affinity of this region to HIF binding. Such an interesting feature of the HIF DNA binding sequence was previously described for the *transferrin* gene enhancer [76]. In fact, in this same study, the authors showed that a single HBS derived from the *Epo* gene was not sufficient to induce expression of a reporter gene, and that concatamerized HBSs were necessary to achieve it. They argued that a single HBS in isolation is not sufficient to convey full hypoxic activation of oxygen-regulated genes and that it requires additional *cis*-acting elements. Such elements differ between genes and could be a CACA box described for the *Epo* [55] and *VEGF* genes [77], an activator protein (AP-1) site [78], a cAMP-responsive element [79], but also the HIF site itself.

On the other hand, we have shown that the labeled −966(G) oligonucleotide was not able to bind alone HIF- 1 in EMSA experiments. However an excess of cold oligonucleotide −966(G) competed both with the TFHBS and +281 HRE. Moreover, in luciferase assays, the presence of −966(A) (−966(A) WTEx2 construct) instead of −966 (G) (−966(G) WTEx2 construct) within the 1.4kb-Exon2 construct, provided a significantly lower luciferase activity upon DFX treatment. These results indicate that, although alone it was not able to bind HIF, the −966(G) HBS plays a role in *HLA-G* gene regulation in hypoxia-mimicking environment.

Collectively, these findings strongly suggest that the primary HIF-1 binding site is the +281 HRE, and that it might connect with the transcriptional complex at the promoter of *HLA-G* by DNA looping, thus rendering the −966 site accessible for HIF-1 binding. DNA looping was previously documented over large distances, making transcriptional activity independent of physical distance along chromosomes [80–83]. Thus −966 G/A polymorphism could contribute to the magnitude of HLA-G expression [84] during pregnancy and cancer, more particularly glioblastoma.

We observed that the amount of HLA-G transcription and protein expression was predominantly controlled by DNA demethylation processes. This result is compatible with previous findings on the role of DNA methylation in HLA-G expression in tumor cells [45, 85–87]. However, it is striking that the effect of DFX was strong enough to still be observed when cells were cultured in medium containing both hypoxia-mimicking and demethylating agents. In agreement with the influence of epigenetics in HIF-mediated response, it is demonstrated the importance of the basal transcriptional permissive state of the genes under normal growth conditions, notably RNA Pol II occupancy on the promoter, and/or histone modifications [72, 88]. Consistently, it is documented that DNA demethylation, as hypoxia, is a key process during pregnancy [89–91] and cancer [92, 93]. Global DNA hypomethylation occurs in the early embryo that expresses HLA-G [94]. On the contrary, an overall progressive increase in average methylation is observed from first to third trimester placenta [95, 96] with a decrease in HLA-G expression, while global hypomethylation has been associated with tumor progression [97, 98]. In addition to the effect of changes in chromatin structure, HREs contain CpG dinucleotides that are potentially methylated, and HREs could become functional following 5-aza-dC treatment. Recent reports showed that HIF binds HREs that are hypomethylated in genes where DNA methylation has been documented as an important gene modulation mechanism [99]. Interestingly, following 5-aza-dC treatment of ovarian cancer cells (BG-1) *HLA-G* −242 site was shown to remain methylated, while hypomethylation of sequences was observed within 5′ *HLA-G* regulatory region [85].

In conclusion, our results support that HLA-G could be involved in glioblastoma evasion to antiangiogenic therapy [100, 101], since *de novo* acute hypoxic stress will lead to HIF-1 stabilization and then binding to the exon 2 HRE. In such a situation −966 G/A polymorphism could modulate the magnitude of HLA-G expression and thus should be taken into account. Besides, 5-aza-dC treatment has been proposed to develop tumor antigen specific active immunotherapy or to reduce telomerase expression in glioblastoma [5]. In such a situation, resident hypoxia and DNA demethylation acting both at the *HLA-G* promoter region and HRE sites, might provide favorable conditions for ectopic HLA-G expression and then tumor invasion.

## MATERIALS AND METHODS

### Cell line and culture

The U251MG glioblastoma cells (kindly provided by Dr H. Wiendl, Department of Neurology, University of Wuerzburg, Wuerzburg, Germany) were maintained in DMEM (Invitrogen Life Technologies, Carlsbad, CA) supplemented with 10% FBS (Sigma-Aldrich, Saint Louis, MO), Gentamycin at 0.02 mg/ml and Fungizone Amphotericin B at 0.25 μg/ml.

Cells were grown in the appropriate medium with hypoxia-mimicking conditions provided by the presence of desferrioxamine (DFX) (Sigma-Aldrich) at 200 μM and 400 μM [2, 53]. (Supplementary Figure S1). Experiments combining both hypoxia-mimicking conditions and DNA demethylation were conducted by treating cells with 5-aza-dC (Sigma-Aldrich) at 100 μM during 72 h and then DFX at 400 μM (concentration providing significant *HLA-G* upregulation) during 24 h.

### Plasmids and transfections

Constructions with the promoterless pGL3 basic vector were performed as previously described [45] using 1438 bp of *HLA-G* promoter region 5′ to the ATG initiation codon (1.4 kb-5′UTR construction). The DNA fragments covering both the 1.4 kb *HLA-G* region and a region spanning ATG to 87 bp of intron 2 (1.4 kb-Exon2 construction) were obtained by PCR using the following specific reverse oligonucleotide 5′-TTTTAGATCTCAGA CCCGGAGACTCGGGAG-3′ that contains a BglII restriction site. Mutated HBSs were introduced within the 1.4 kb-Exon2 construction using complementary mutated oligonucleotides carrying the same mutations as in EMSA. Site-directed mutagenesis of the two HBSs in exon 2 was performed by MilleGen (Labège, France). In all luciferase assays, the cells were co-transfected with the pRL-TK Renilla Luciferase reporter vector (Promega, Charbonnières-les-Bains, France) used as a control of the variations of the transfection efficiency and protein extracts preparation.

The HIF-1α shRNA plasmid and the control shRNA Plasmid A (Sc-44225 and Sc-108060, Santa Cruz Biotechnology, Inc, Santa Cruz, CA) were used to silence HIF-1α expression in U251MG cells according to manufacturer's instructions. Briefly, transfections with shRNA plasmids were facilitated by the Lipofectamin 2000 reagent (Invitrogen Life Technologies). The Lipofectamin-DNA mixture was added to the cells incubated in serum and antibiotic free medium that was replaced by supplemented medium after 4 h. In transient expression, the assays were executed 48 h after transfection. In stable expression, cells were cultured in the medium supplemented with Puromycin (Invivogen, Toulouse, France) at 3.5 μg/ml.

### QRT-PCR analysis

Total RNA was extracted using TRIzol^®^ reagent (Invitrogen Life Technologies) according to the manufacturer's instructions. Residual DNA was eliminated by DNAse I treatment (New England Biolabs, Evry, France) for 10 min at 37°C. 5 μg of total RNA from each sample was retrotranscribed to cDNA with Moloney murine leukemia virus (M-MLV)-Reverse Transcriptase (Invitrogen Life Technologies) at 42°C during 1 h. 0.5 μg of cDNA samples was used as template for amplification reactions carried out with the TaqMan real-time PCR assay in an ABI 7000 sequence detection system (Applied Biosystems Life Technologies, Villebon sur Yvette, France). For each experiment, mixed PCR reaction has been performed in duplicates, in the presence of GAPDH specific oligonucleotides as an internal standard (Applied Biosystems Life Technologies), HLA-G specific primers 5′-CTGGTTGTCCTTGCAGCTGTAG-3′ (forward) and 5′-CCTTTTCAATCTGAGCTCTTCTTTCT-3′ (reverse) (Eurogentec, Seraing, Belgium) and the TaqMan TAMRA probe 5′-CACTGGAGCTGCGGTCGCTGCT-3′ (Applied Biosystems Life Technologies). Quantification relative to HLA-G^+^ JEG-3 (assigned a value of 1) was determined by the ΔΔCt method [10].

HIF-1α expression analysis was assessed using specific primers 5′-CCTTCGATCAGTTGTCACCA TTAGA-3′ (reverse) 5′-ACTGTAACTGTGCTTTGAGGA CTTG-3′ (forward) and the TaqMan FAM probe 5′-CAGTTCCGCAAGCCCT-3′ (Applied Biosystems Life Technologies). GAPDH was used as a reporter gene (Applied Biosystems Life Technologies). Each experiment was repeated at least three independent times.

### Protein extraction and western blot

Protein extracts from cells, either cultured or not with DFX and/or 5-aza-dC, were isolated using the ProteoJET Cytoplasmic and Nuclear Protein Extraction kit (Fermentas Thermo Fisher Scientific, Villebon sur Yvette, France), in the presence of protease and phosphatase inhibitors according to manufacturer's instructions. The extracts were quantified using the BCA Protein Assay kit (Pierce Thermo Fisher Scientific, Illkirch, France). Proteins were then separated on 4 %–8 % or 4 %–12 % gradient polyacrylamid gels in TG-SDS 1× Buffer. Proteins were transferred on a Hybond-C Extra membrane (Amersham Biosciences, Velizy-Villacoublay, France) in TG 1x-Ethanol 20% Buffer. The membranes were blocked for 30 min in 5% non-fat milk in phosphate buffered saline (PBS) containing 0.2 % Tween 20, and incubated with the primary antibody overnight at 4°C: HIF-1α (BD Transduction Laboratories), HLA-G 4H84 clone at 1 μg/ml (Exbio, Praha, Czech Republic), and β-actin at 0.5 μg/ml (Sigma-Aldrich). After washing in PBS-Tween, membranes were incubated for 1 h at room temperature with peroxidase-labeled secondary antibodies. The immunoreactive bands were visualized by chemiluminescence (Amersham Biosciences).

### Electrophoretic mobility shift assay (EMSA)

γ-^32^P (Amersham) double stranded oligonucleotides (Eurogentec) encompassing the HREs of *HLA-G* and *transferrin* (control) were as follow (only the top strands are shown; HBSs are underlined): *HLA-G* promoter region at −966 bp 5′-TAAAAACAGGCAGTGCG TGAGCACTAGTGAGGGG-3′ and −242 bp 5′-TCCCAG GGCCTCAAGCGTGGCTCTCA-3′ [53]; *HLA-G* exon 2 at +281 bp and +291 bp 5′-CATGGGCTACGTGGACGAC ACGCAGTTCGT-3′, and a consensus HBS from *transferrin* gene promoter TFHBS 5′-TTCCTGCACGTAC ACACAAAGCGCACGTATTTC-3′ [76]. Oligonucleotides which allowed us to create a uniquely located abasic site in the duplex at the HRE binding contained a dU replacing a G in the HBS: −966 bp F (Forward) abasic 1 5′-TAAAAACAGGCAGTdUCGTGAGCACTAGTGA GGGG-3′, −966 bp F abasic 2 5′-TAAAAACAGGCAGTG CGTdUAGCACTAGTGAGGGG-3′, and −242 bp F abasic 5′-TCCCAGGGCCTCAAGCGTdUGCTCTCA-3′, +281 bp F abasic 5′-CATGGGCTACGTdUGACGACACGCAGT TCGT-3′ and +281 bp R (Reverse) abasic 5′-ACG AACTGCGTdUTCGTCCACGTAGCCCATG-3′). The dU-containing oligonucleotide annealed to its wild type complement and abasic sites were generated by treating duplexes with Uracil N-glycosylase (New England Biolabs) during 30 min.

Nuclear extracts were incubated alone for 15 min at room temperature in Darnell binding buffer (KCl 400 mM, Hepes pH 7.5 200 mM, MgCl_2_ 10 mM, EGTA 1 mM, DTT 5 mM and Ficoll 4%). Binding reactions were carried out by incubating nuclear extracts (2–4 μl) with radiolabeled oligonucleotide probes (1–2 ng; 1.5 × 10^5^ cpm) for further 20 min at room temperature in the presence of 2 μg/μl sonicated salmon sperm DNA (Invitrogen Life Technologies) to reduce nonspecific binding. Competition experiments were assessed by adding a 100-fold molar excess of unlabeled double stranded oligonucleotides corresponding to irrelevant (5′-GCTCCTTCTGAGTATCTTTACA-3′), specific competitors and oligonucleotides with mutation introduced at the HBS: −966(A) 5′-TAAAAACAGG CAGTGCATGAGCACTAGTGAGGGG-3′, +281 mut 5′ 5′-CATGGGCTAAAAGGACGACACGCAGTTCGT-3′, +281 mut 3′ 5′-CATGGGCTACGTGGACGACTTTCA GTTCGT-3′ and +281 mut 5′-CATGGGCTAAAAGGAC GACTTTCAGTTCGT-3′). In order to identify HIF-1α and HIF-2α in the DNA-Protein complexes, supershift experiments were conducted with 2 μg of rabbit anti-HIF- 1α (sc-10790, Santa Cruz Biotechnology) and anti-HIF-2α (ab199, Abcam, Paris, France) antibodies incubated with the nuclear extracts before the addition of radiolabeled probe.

The DNA-Protein complexes were then subjected to electrophoresis on a 3 % non-denaturing polyacrylamide gel at room temperature in 0.5X TBE at 200 V for 2 h, and processed for autoradiography.

### Luciferase reporter assays

Reporter assays were conducted using the Dual Luciferase Reporter Assay System (Promega) according to the manufacturer's instructions. Aliquots of cell lysates were transferred into a 96 well microplate and incubated successively with the Luciferase and Renilla substrates. Luminescence was recorded using the FluoStar Optima plate reader (BMG Labtech, Champigny sur Marne, France) for each reaction on the same aliquot. Luciferase activities were then corrected by normalization to the corresponding Renilla activities, to control the variations of the transfection efficiency and protein extracts preparation. All assays were performed in duplicates from three independent experiments.

### DNA methylation analysis by pyrosequencing

Quantitative DNA methylation analysis was performed by pyrosequencing of bisulfite treated DNA [102]. One μg of DNA was bisulfite converted using the EpiTect 96 Bisulfite kit (Qiagen, Hilden, Germany) according to the manufacturer's instructions. Regions of interest for validation were amplified using 30 ng of bisulfite treated human genomic DNA and 5 to 7.5 pmol of forward and reverse primer, one of them being biotinylated. Sequences for oligonucleotides for PCR amplification and pyrosequencing are indicated in Table 2. Reaction conditions were 1x HotStar Taq buffer supplemented with 1.6 mM MgCl2, 100 μM dNTPs and 2.0 U HotStar Taq polymerase (Qiagen) in a 25 μl volume. The PCR program consisted of a denaturing step of 15 min at 95°C followed by 50 cycles of 30 s at 95°C, 30 s at the respective annealing temperature and 20 s at 72°C, with a final extension of 5 min at 72°C. 10 μl of PCR product were rendered single-stranded as previously described [103] and 4 pmol of the respective sequencing primer were used for analysis. Quantitative DNA methylation analysis was carried out on a PSQ 96 MD system with the PyroGold SQA Reagent Kit (Qiagen) and results were analyzed using the PyroMark CpG software (V.1.0.11.14, Qiagen).

**Table 2:** Primers used in quantitative DNA methylation analysis performed by pyrosequencing of bisulfite treated U251MG DNA

Gene	Size	PCR primer forward	PCR primer reverse	Pyrosequencing primer(s)	CpGs
HLA-G_AMP1 chr6:29794412-29794951	114	Biotin-TGGATATTTTTT AAAAATAGGTAGTG	TATTACAACCAAAAA CCAACACAAA	TCAATACAATCA CAATACCC	1
HLA-G_AMP2 chr6:29795132-29795671	222	GATTTAGGGAGA TATTGAGATAGAA	Biotin-CACCTAATAAAAA TAAAAACTAAAACC	GTTTGGTATAAGA GTAG	1–7
HLA-G_AMP3 chr6:29795612-29796151	234	GTTTTTATTTTATG AGGTATTTTAG	Biotin-ACAAATTCATTCT ATCAATCTATAC	TTTATTTTATGAGG TATTTT	1–21
				GGAGTAGGAGGGGT	22–24

### Chromatin immunoprecipitation assays (ChIP)

To analyze *in situ* the binding of HIF-1α to the HLA-G locus we used CHIP-IT Express HT Assays (Active Motif, La Hulpe, Belgium) according to the manufacturer's recommendation. Briefly, formaldehyde cross-linked chromatin from DFX- and 5-aza-dC-treated U251MG cells was sonicated and immunoprecipitated with either anti-HIF-1α antibody (NB100-105, Novus Biologicals, Littleton, CO, USA) or control Rabbit IgG, and protein G-coated magnetic beads. Immunoprecipitated DNA was amplified using specific exon 2 *HLA-G* primers (G25′AAV Forward: 5′-TCCATGAGGTATTTCAGCGC and G.257 Reverse: 5′-TGTTCCGTGTCTCCTCTTCC) and visualized with Ethidium Bromide coloration after agarose gel migration.

### Statistical analysis

GraphPad Prism was used for statistical analysis. Data are presented as mean ± SEM and statistical analysis was performed for at least three independent experiments using Mann-Whitney *U* test or Wilcoxon matched-pairs signed rank test. A *p* value < 0.05 was considered significant.

## SUPPLEMENTARY MATERIALS FIGURE


