# Supplementary material for: Hypoxia inducible factor-1 mediates the expression of the immune checkpoint HLA-G in glioma cells through hypoxia response element located in exon 2
Source: Oncotarget. 2016 Aug 26;7(39):63690–707. doi: 10.18632/oncotarget.11628 (PMC5325396; doi:10.18632/oncotarget.11628)
Supplement: Supplementary file 1 [file oncotarget-07-63690-s001.pdf]

Hypoxia inducible factor-1 mediates the expression of the immune checkpoint HLA-G in glioma cells through hypoxia response element located in exon 2

Supplementary Materials

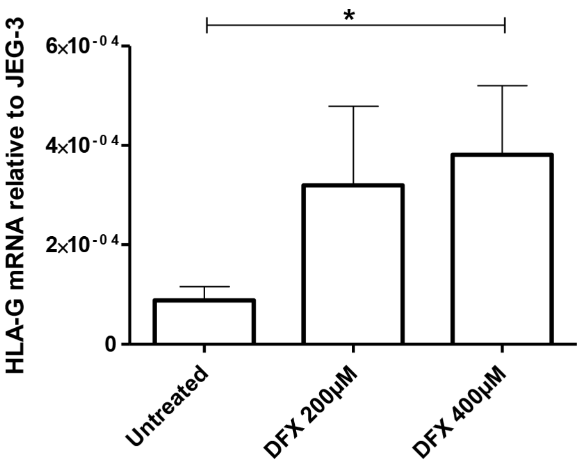

Supplementary Figure S1: Significant induction of *HLA-G* expression in U251MG cells treated with 400 µM DFX. Real-time RT-PCR analysis, targeting all HLA-G mRNA forms, carried out on cells cultured with normal growth conditions (Untreated) or treated with DFX at 200 µM and 400 µM for 24 h (4 independent experiments in duplicates). Data is presented as mean ± SEM and statistical analysis was performed using Mann-Whitney *U* tests (* indicates a p value < 0.05).
